# Overexpression of *OsPIN9* Impairs Chilling Tolerance via Disturbing ROS Homeostasis in Rice

**DOI:** 10.3390/plants12152809

**Published:** 2023-07-28

**Authors:** Qiqi Ouyang, Yanwen Zhang, Xiaoyi Yang, Chong Yang, Dianyun Hou, Hao Liu, Huawei Xu

**Affiliations:** College of Agriculture, Henan University of Science and Technology, Luoyang 471000, China; 17538391647@163.com (Q.O.); 18438616118@163.com (Y.Z.); yangxy@163.com (X.Y.); yc051722@163.com (C.Y.); dianyun518@163.com (D.H.); liuhao_1990@126.com (H.L.)

**Keywords:** *OsPIN9*, polar auxin transport, chilling tolerance, ROS homeostasis, antioxidant enzymes, rice (*Oryza sativa* L.)

## Abstract

The auxin efflux transporter PIN-FORMED (PIN) family is one of the major protein families that facilitates polar auxin transport in plants. Here, we report that overexpression of *OsPIN9* leads to altered plant architecture and chilling tolerance in rice. The expression profile analysis indicated that *OsPIN9* was gradually suppressed by chilling stress. The shoot height and adventitious root number of *OsPIN9-*overexpressing (OE) plants were significantly reduced at the seedling stage. The roots of OE plants were more tolerant to *N*-1-naphthylphthalamic acid (NPA) treatment than WT plants, indicating the disturbance of auxin homeostasis in OE lines. The chilling tolerance assay showed that the survival rate of OE plants was markedly lower than that of wild-type (WT) plants. Consistently, more dead cells, increased electrolyte leakage, and increased malondialdehyde (MDA) content were observed in OE plants compared to those in WT plants under chilling conditions. Notably, OE plants accumulated more hydrogen peroxide (H_2_O_2_) and less superoxide anion radicals ($O_{2}^{-}$) than WT plants under chilling conditions. In contrast, catalase (CAT) and superoxide dismutase (SOD) activities in OE lines decreased significantly compared to those in WT plants at the early chilling stage, implying that the impaired chilling tolerance of transgenic plants is probably attributed to the sharp induction of H_2_O_2_ and the delayed induction of antioxidant enzyme activities at this stage. In addition, several *OsRboh* genes, which play a crucial role in ROS production under abiotic stress, showed an obvious increase after chilling stress in OE plants compared to that in WT plants, which probably at least in part contributes to the production of ROS under chilling stress in OE plants. Together, our results reveal that *OsPIN9* plays a vital role in regulating plant architecture and, more importantly, is involved in regulating rice chilling tolerance by influencing auxin and ROS homeostasis.

## 1. Introduction

Due to extreme weather events, plants are progressively subjected to various abiotic stresses, such as chilling stress. Chilling stress restricts the geographical distribution and affects plant productivity [1,2]. Rice (*Oryza sativa* L.) originates from tropical and subtropical regions and is more sensitive to chilling stress than other cereal crops [3,4,5]. Previous data showed that rice yield decreased by 30–40% due to cold stress in temperate areas [6]. Therefore, improving rice chilling tolerance for maintaining food security is an urgent target [7]. In recent years, excellent progress has been made in understanding the physiological and molecular mechanisms underlying chilling stress [1,8,9,10]. Chilling stress triggers Ca^2+^ influx, which plays a key role in chilling tolerance [1,11,12,13]. For example, COLD1 (CHILLING-TOLERANCE DIVERGENCE 1) and OsCNGC9 (CYCLIC NUCLEOTIDE-GATED CHANNEL 9) have been demonstrated to regulate rice chilling tolerance by mediating Ca^2+^ influx under chilling stress [7,14]. Growing evidence shows that low temperatures dramatically induce the expression of the well-known C-repeat binding factor (*CBF*)/dehydration-responsive element binding factor (*DREB*) genes, which play a crucial role in plant chilling tolerance [15,16,17,18]. Conversely, evidence also showed that the transcriptional abundance of these transcription factors plays a minor role in cold tolerance [19,20,21]. Additionally, reactive oxygen species (ROS) are pivotal in various abiotic stress adaptations [22,23]. Low-level ROS at the early stress stage act as signals to trigger diverse stress responses, whereas excessive ROS cause cell damage; therefore, ROS homeostasis should be well controlled during abiotic stresses [10], including chilling tolerance [21,24,25,26]. In line with this, it is evidenced that more ROS are accumulated at the relative earlier chilling stage, while less ROS accumulation is detected at the late chilling stage in high chilling tolerance rice than in low chilling tolerance rice, which suggests that ROS homeostasis plays a vital role in regulating rice chilling tolerance [21]. 

The phytohormone auxin (indole-3-acetic acid, IAA) functions in almost all plant developmental processes and is involved in responses to many environmental clues, such as light, gravity, and temperature fluctuations [27,28,29,30]. Most auxin is synthesized in primordial and young leaves [31,32,33] and is transported basipetally to facilitate plant growth and development. The plant-specific PIN-FORMED (PIN) efflux carriers are the most important players in this process [34], which cooperated with the AUXIN1 (AUX1)/LIKE AUX1 (LAX) influx carriers, the phosphoglycoprotein (PGP/MDR/ABCB) efflux/influx transporters [35,36] and PIN-LIKES (PILS) family proteins [37] to drive polar auxin transport. 

Significant progress has been made in the role of *PIN* genes, which are mainly derived from the dissection of *PIN* genes in the model plants *Arabidopsis thaliana* and rice [38,39,40,41,42]. The *Arabidopsis* genome possesses eight *PIN* genes, and the function of these *AtPIN* genes has been deeply investigated [38,43,44]. In contrast, the role of rice *OsPIN* genes is largely unknown. Twelve *OsPIN* genes have been identified in the rice genome [45,46], and several *OsPIN* genes have been functionally identified to play key roles in regulating rice growth and development by influencing polar auxin transport. *OsPIN1b*, which is also named *OsPIN1*, is mainly expressed in the vascular tissues and root primordial and functions in regulating the emergence of adventitious roots [47]. Our previous study showed that *OsPIN1b*, also designated as *OsPIN1a* [46], is associated with root negative phototropism [48]. Further investigation indicated that *OsPIN1a* and *OsPIN1b* redundantly regulate root and inflorescence development, while *OsPIN1c* and *OsPIN1d* are redundantly involved in modulating panicle formation [49]. *OsPIN2* regulates rice root system architecture and gravitropism by influencing auxin transport and distribution in roots, especially in columella cells [50,51,52]. *OsPIN5b* is constitutively expressed in vegetative and reproductive tissues and functions in modulating rice architecture and yield [53]. *OsPIN10a* (also designated as *OsPIN3t*) is mainly expressed in vascular tissue and functions in drought stress response and drought tolerance [54]. Recently, the monocot-specific *OsPIN9* gene has been indicated to function in regulating tiller bud outgrowth and affecting tiller number [55]. In addition, the expression profile of *PIN* genes, such as tissue-specific analysis and response to abiotic stress, in the other two monocot plants, sorghum (*Sorghum bicolor*) and maize (*Zea mays*), was deeply investigated [56,57,58]. Taken together, although excellent progress has been made in the functional dissection of *OsPIN* genes, which mainly focuses on rice growth and development, whether and how *OsPIN* genes are involved in regulating abiotic stresses, especially chilling stress, is still elusive.

The underlying mechanism of auxin in regulating cold tolerance is largely unknown, although auxin plays key roles in regulating plant growth and development. Increasing evidence has shown that cold stress is tightly related to auxin homeostasis. For example, an earlier study indicated that temperature could influence exogenous auxin transport velocity in some plant species [59]. Although the underlying mechanism of auxin in regulating cold tolerance is almost completely unknown, cold stress not only differentially regulates the expression of auxin-responsive genes but also markedly increases auxin content in rice [30,60]. Additionally, auxin transport could be suppressed and restored by low temperature and room temperature, respectively [61]. Inhibition of intracellular trafficking of auxin efflux carriers substantially influences auxin homeostasis under cold conditions [29]. Further research demonstrated that GNOM, a SEC7 containing ARF-GEF, which is closely associated with endosomal trafficking of auxin efflux carriers, can positively regulate *Arabidopsis* cold tolerance [62]. Collectively, these results strongly suggest that auxin homeostasis and transport are implicated in regulating plant chilling tolerance. However, little is known about the underlying molecular mechanisms involved in the role of auxin homeostasis and transport in chilling stress.

Previous studies demonstrated that *OsPIN9* functions in polar auxin transport and is involved in regulating adventitious root number and tiller number [55]. Recently, Manna et al. (2022) [63] reported that salt and drought treatment significantly induced *OsPIN9* expression, indicating the potential role of *OsPIN9* in abiotic stress adaptation. In line with this, our previous study showed that mutation of *OsPIN9* by CRISPR/Cas9 technology confers enhanced chilling tolerance in rice [64]. However, whether and how *OsPIN9* overexpression regulates rice chilling tolerance is unknown. In addition, since off-target effects or newly formed mutation proteins derived from CRISPR/Cas9 technology have the potential to cover the true function of target genes, it is necessary to further verify gene function by upregulating its expression. Here, we functionally examined the role of *OsPIN9* in modulating chilling tolerance by overexpressing technology. Our results showed that overexpression of *OsPIN9* not only leads to the alteration of rice architecture but also impairs rice chilling tolerance. We provided strong evidence that *OsPIN9* plays a crucial role in modulating rice chilling tolerance and shed light on the relationship between polar auxin transport and chilling tolerance in rice.

## 2. Results

### 2.1. Expression Patterns of OsPIN Genes under Chilling Stress

**Figure 1:**
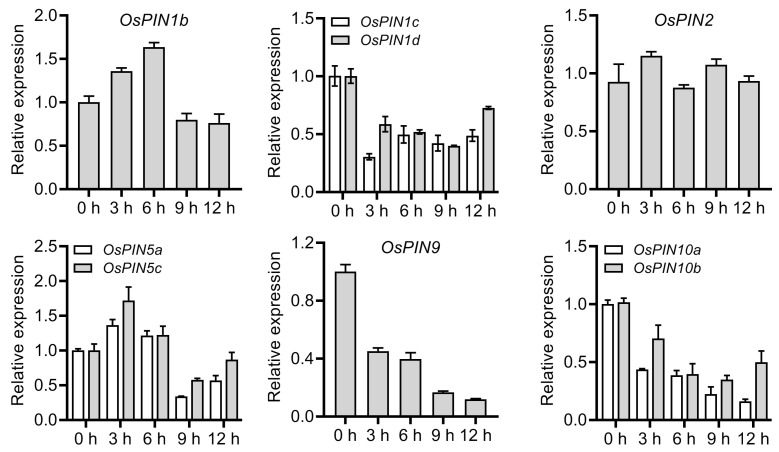
Expression profiles of *OsPIN* genes under chilling treatment. Values are means ± standard deviation (SD) (*n* = 3).

Increasing evidence shows that plant chilling tolerance is tightly associated with polar auxin transport [29,59,61,62]. However, the mechanism by which *OsPIN* genes respond to chilling stress is still largely unknown. To this end, quantitative real-time polymerase chain reaction (qRT-PCR) was performed to evaluate the responses of *OsPIN* genes in rice roots upon chilling stress. Nine *OsPIN* genes (*OsPIN1b*, *OsPIN1c*, *OsPIN1d*, *OsPIN2*, *OsPIN5a*, *OsPIN5b*, *OsPIN9*, *OsPIN10a*, and *OsPIN10b*), which can be detected in rice roots [39,45,46], were selected to analyze the expression profiles under chilling stress. *OsPIN1b* displayed a different expression pattern under chilling treatment compared to *OsPIN1c* and *OsPIN1d*, albeit belonging to the same subfamily. In detail, *OsPIN1b* was induced to the highest expression after chilling for 6 h and then decreased sharply, while *OsPIN1c* and *OsPIN1d* were suppressed after chilling for 3 h and kept to a relatively constant level thereafter. *OsPIN2* remained at a constant level under chilling treatment. Similar to *OsPIN1b*, *OsPIN5a* and *OsPIN5c* also showed increased expression first and then decreased. Three monocot-specific *PIN* genes, *OsPIN9*, *OsPIN10a*, and *OsPIN10b*, were greatly inhibited after chilling treatment and gradually decreased with treatment duration (Figure 1). Intriguingly, the paralogous *OsPIN* genes, *OsPIN1c*/*1d*, *OsPIN5a*/*5c*, and *OsPIN10a*/*10b*, showed a similar expression profile under chilling stress, implying that they might play a synergistic role in chilling tolerance.

### 2.2. Generating OsPIN9-Overexpressing Transgenic Rice and Phenotypes of Transformants

**Figure 2:**
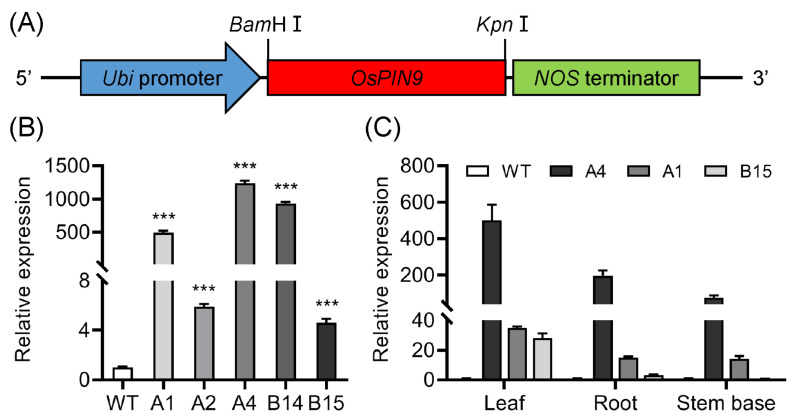
Generation of *OsPIN9*-overexpressing transgenic lines. (**A**) Schematic diagram of *pUbi*:*OsPIN9*. (**B**) Expression of *OsPIN9* in different transgenic lines. The expression level of *OsPIN9* in WT was set to one. The data were analyzed by ANOVA and Tukey’s tests at a *p* < 0.05 significance level. ***: *p* < 0.001. (**C**) Expression analysis of *OsPIN9* in diverse tissues of *OsPIN9* transgenic lines. Values are means ± standard deviation (SD) (*n* = 3).

The monocot-specific *OsPIN9*, which was dramatically suppressed under chilling treatment, was chosen for further study. To obtain insight into the function of *OsPIN9* in chilling tolerance, a plasmid carrying the *OsPIN9* ORF, driven by a strong constitutive *Ubiquitin* promoter (*pUbi*) (Figure 2A), was introduced into rice cultivar Nipponbare by *Agrobacterium*. The transgenic plants were confirmed by PCR using genomic DNA as a template with specific primers. qRT-PCR analysis showed that the *OsPIN9* transcript significantly increased by 3.6–1237 times in the five tested transgenic line roots compared with that in WT plants (Figure 2B). Among which, A4 and B14 represented the high expression levels, A1 represented the moderate expression level, and A2 and B15 represented the low expression levels. Three lines, A4, A1, and B15, which represented high, moderate, and low expression levels of *OsPIN9*, were employed to further analyze the expression of *OsPIN9* in leaves, roots, and stem bases. The highest increase in expression was detected in leaves, followed by roots and stem bases (Figure 2C). Two T_2_ homozygous transgenic lines, A4 and B14, with high expression levels of *OsPIN9*, designated as OE1 and OE2, respectively, were used for further investigation.

**Figure 3:**
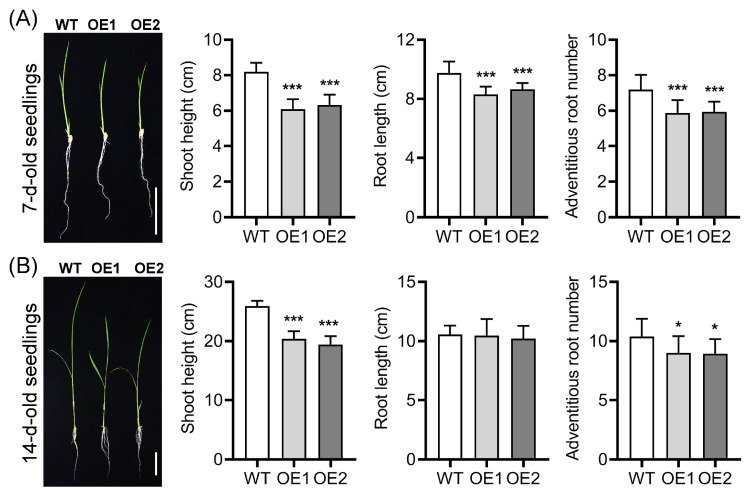
Phenotypes of wild-type (WT) and *OsPIN9*-overexpressing lines (OE) in 7 day old seedlings (**A**) and 14 day old seedlings (**B**). Bar = 4 cm. Values are means ± standard deviation (SD) (*n* = 24). The data were analyzed by ANOVA and Tukey’s tests at a *p* < 0.05 significance level. *: *p* < 0.05; ***: *p* < 0.001.

Overexpression of *OsPIN9* significantly decreased shoot height, root length, and adventitious root number after germination for 7 d in comparison with those in WT plants (Figure 3A). In detail, compared with the WT plants, the shoot height, root length, and adventitious root number of the OE plants were significantly decreased by 23–26%, 11–15%, and 17–18%, respectively. After germination for 14 d, shoot height and adventitious root number of OE lines declined by 21–25% and 13–14%, respectively, compared to those in WT plants. However, the root length was similar to WT plants at this stage (Figure 3B), indicating that rice plants can dynamically adapt to the change in auxin transport to some extent.

### 2.3. Overexpression of OsPIN9 Disturbs Auxin Homeostasis in Rice

**Figure 4:**
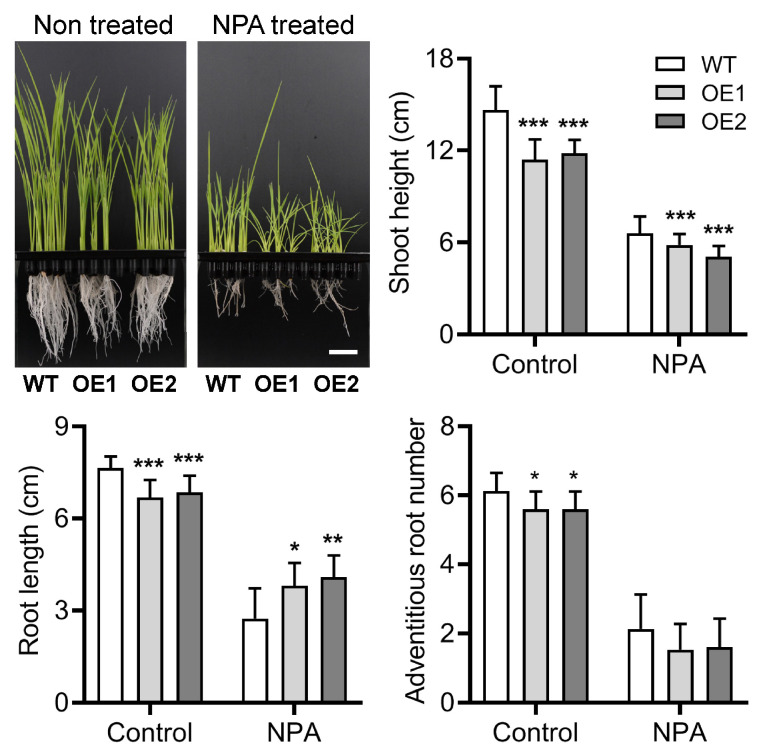
Sensitivity analysis of wild-type (WT) and *OsPIN9*-overexpressing (OE) plants under NPA treatment. Bar = 2 cm. Values are means ± standard deviation (SD) (*n* = 24). The data were analyzed by ANOVA and Tukey’s tests at a *p* < 0.05 significance level. *: *p* < 0.05; **: *p* < 0.01; ***: *p* < 0.001.

It was reported that overexpression of *OsPIN9* accelerated auxin transport from shoots to roots and resulted in an increase and decrease of IAA in roots and shoots, respectively [55]. *N*-1-naphthylphthalamic acid (NPA) is an inhibitor of polar auxin transport and can interact with PIN carriers directly [65]. To further assess the role of *OsPIN9* in auxin transport, germinated wild-type and transgenic seeds were cultured in Kimura B complete nutrient solution [39] supplemented with 0.5 μM NPA. After 7 d treatment, NPA obviously inhibited plant growth; the shoot height of OE lines was significantly decreased compared to WT plants both under normal conditions and NPA treatment. Contrastingly, the root length of OE lines was shorter than that of WT under normal conditions, while it increased significantly more than that of WT plants under NPA treatment. In detail, the root length of OE lines was significantly decreased by 10–12% when compared with WT under normal conditions, whereas it was about 39% and 49% longer compared with control plants after NPA treatment, indicating the roots of OE lines were more resistant to NPA than those of WT plants (Figure 4). Overexpression of *OsPIN9* significantly lowered the adventitious root number under control conditions, while NPA treatment severely decreased the adventitious root number and completely abolished the difference in adventitious root number between WT and OE lines. In addition, the ability to grow upright in some OE plant shoots was obviously inhibited and showed a partially impaired negative gravitropism compared with that in WT plants, indicating that the shoots of OE lines are probably more sensitive to NPA than those of WT plants (Figure 4). Collectively, these results demonstrate that auxin homeostasis is disturbed in OE lines.

### 2.4. Overexpression of OsPIN9 Impairs Chilling Tolerance in Rice

**Figure 5:**
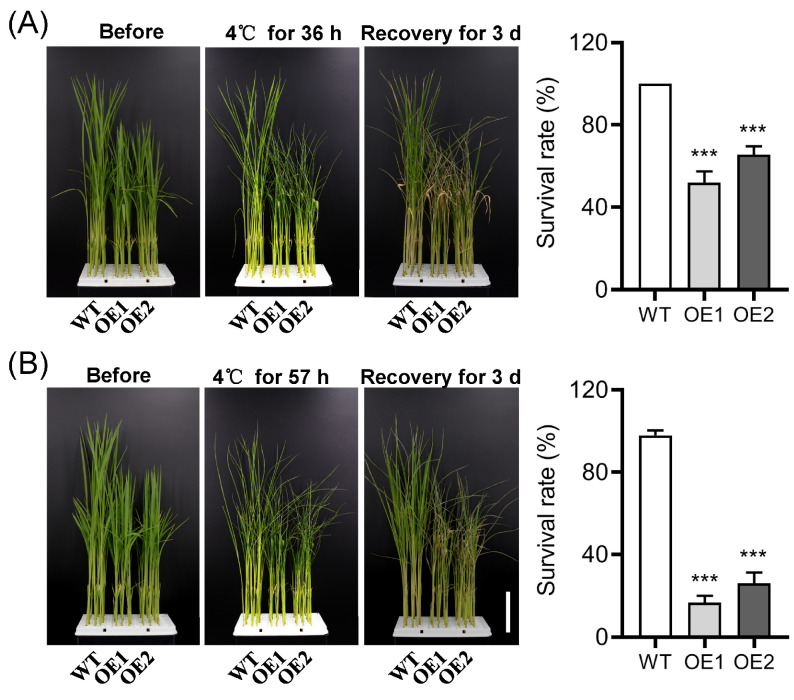
Chilling tolerance analysis of WT and *OsPIN9*-overexpressing (OE) rice. Wild-type (WT) and OE plants were treated with 4 °C for 36 h (**A**) and 57 h (**B**), followed by another 3 days of recovery under normal conditions, and then the survival rate was analyzed statistically. Bar = 5 cm. Values are means ± standard deviation (SD) (*n* = 24). The data were analyzed by ANOVA and Tukey’s tests at a *p* < 0.05 significance level. ***: *p* < 0.001.

Due to the fact that polar auxin transport is closely related to chilling stress [66], and PIN carriers perform a rate-limiting role in regulating auxin efflux [34], as well as the quick suppression of *OsPIN9* under chilling stress (Figure 1), the role of *OsPIN9* in chilling tolerance was evaluated in WT and OE plants. Fourteen day old rice seedlings were moved from 28 °C to 4 °C. After chilling for 36 h, most WT leaves remained normal, but almost all leaves of OE plants displayed rolling and wilting (Figure 5A). The survival rate of OE plants was significantly lower than that of WT plants after 3 d of recovery at 28 °C (Figure 5A). Consistently, after 57 h of chilling treatment followed by 3 d of recovery, the survival rate of WT was dramatically higher than that of OE plants (Figure 5B). In detail, WT plants showed a 98% survival rate, while the survival rates of OE1 and OE2 were only about 17% and 26%, respectively. These results suggest that the expression of *OsPIN9* is involved in negatively modulating rice chilling tolerance.

**Figure 6:**
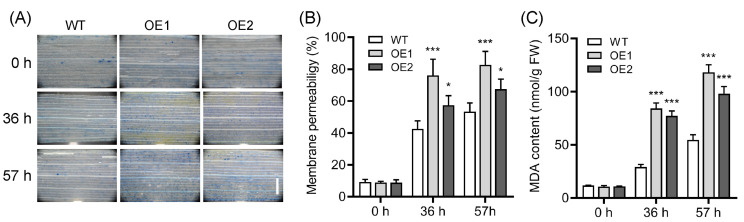
Overexpression of *OsPIN9* causes cell damage in rice seedling leaves. (**A**) Trypan blue staining. Bar = 1 mm. (**B**) Electrolyte leakage. (**C**) Malondialdehyde contents. Values are means ± standard deviation (SD) (*n* = 6). The data were analyzed by ANOVA and Tukey’s tests at a *p* < 0.05 significance level. *: *p* < 0.05; ***: *p* < 0.001.

We then detected several physiological indicators, including cell death, electrolyte leakage, and malondialdehyde (MDA) contents, to evaluate the influence of overexpression of *OsPIN9* on rice chilling tolerance. Trypan blue staining, which is an indicator of cell death, was performed to monitor the cell damage. There was no apparent difference in trypan blue staining in leaves between WT and OE plants under normal growth conditions, while staining was more intensive in the leaves of OE plants compared to that of WT plants after chilling for 36 h and 57 h (Figure 6A), indicating that the cell death in OE plants is greater than that in WT plants. In line with this, another two indicators, electrolyte leakage and MDA contents, were both significantly increased in OE plants compared to those in WT plants after chilling for 36 h and 57 h (Figure 6B,C). Taken together, these results strongly suggest that overexpression of *OsPIN9* substantially impairs rice chilling tolerance.

### 2.5. The Role of OsDREB1 Regulon and Ca^2+^ Signaling-Related Genes in Chilling Stress

**Figure 7:**
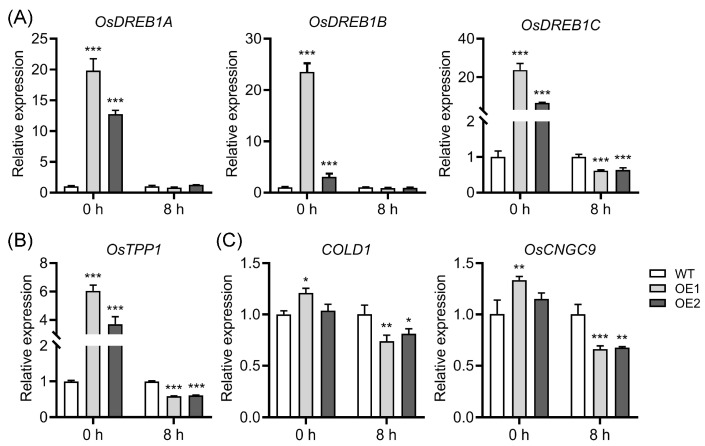
Expression analysis of *OsDREB1* genes, *OsTPP1*, and Ca^2+^ signaling genes under normal and chilling conditions in wild-type (WT) and *OsPIN9*-overexpressing (OE) plants. (**A**) *OsDREB1s* gene expression analysis. (**B**) *OsTPP1* expression analysis. (**C**) Ca^2+^ signaling gene expression analysis. Values are means ± standard deviation (SD) (*n* = 3). The data were analyzed by ANOVA and Tukey’s tests at a *p* < 0.05 significance level. *: *p* < 0.05; **: *p* < 0.01; ***: *p* < 0.001.

The expression of *OsDREB1* genes could be dramatically induced by low temperatures [19,67], and more and more evidence has demonstrated that *OsDREB1* genes play a vital role in chilling stress [14,19,67]. We then detected the expression levels of *OsDREB1A*, *OsDREB1B*, and *OsDREB1C* in WT and OE plants upon chilling stress. Under normal conditions, these *OsDREB1* genes were significantly more induced in OE plants than in WT plants (Figure 7A). However, after chilling for 8 h, the expression levels of these genes in OE plants were similar to or even lower than those in WT plants (Figure 7A). Similarly, trehalose-6-phosphate phosphatase 1 (*OsTPP1*), which has been demonstrated to play a vital role in rice cold tolerance [9,68], displayed a similar expression profile with *OsDREB1* genes in WT and OE plants under both normal and chilling conditions (Figure 7B). Additionally, increasing evidence shows that Ca^2+^ signaling is involved in chilling tolerance regulation [9,69], and *COLD1* and *OsCNGC9* have been demonstrated to facilitate cytoplasmic Ca^2+^ elevation and positively regulate rice cold tolerance [7,14]. To evaluate the role of these Ca^2+^ signaling-related genes in OE lines under chilling stress, we further examined the expression of these two genes under normal and chilling conditions. The results showed that both *COLD1* and *OsCNGC9* in OE lines were slightly induced compared to those in WT plants under normal conditions, while strikingly suppressed under chilling stress (Figure 7C). Collectively, these data suggest that the defense response might be triggered in advance under normal conditions, and the *DREB1* regulon and Ca^2+^ signaling are likely to be implicated in regulating the chilling tolerance of OE lines.

### 2.6. OsPIN9-Overexpressing Plants Accumulated More H_2_O_2_ Rather Than $O_{2}^{-}$ under Chilling Stress

**Figure 8:**
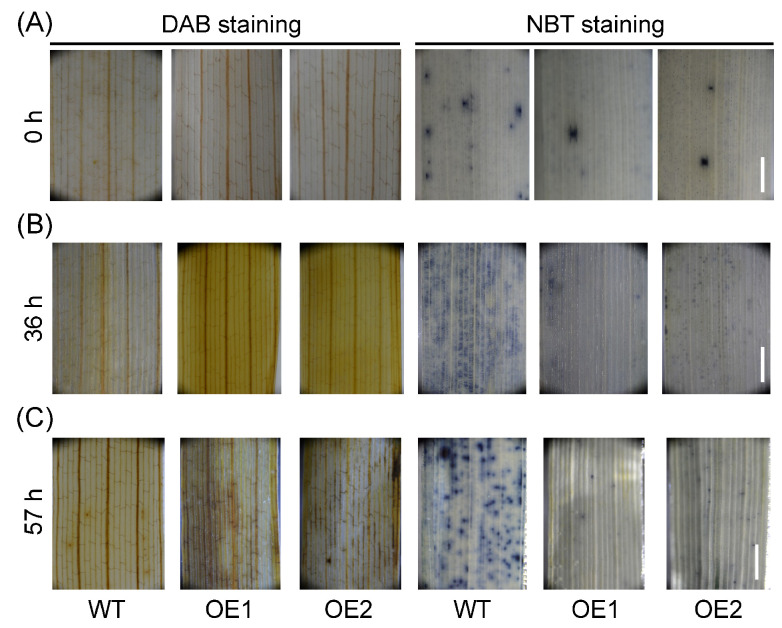
ROS detection in wild-type (WT) and *OsPIN9*-overexpressing (OE) leaves before and after chilling treatments. H_2_O_2_ and $O_{2}^{-}$ in WT and OE leaves were monitored by DAB and NBT staining, respectively, before chilling stress (**A**) and after chilling for 36 h (**B**) and 57 h (**C**). Bar = 1 mm.

In addition to the well-known CBF/DREB regulon, reactive oxygen species (ROS) also play a crucial role in chilling stress [21]. To further investigate the underlying mechanism of *OsPIN9* under chilling stress, ROS content assays were performed by diaminobenzidine tetrahydrochloride (DAB 4HCl) staining for H_2_O_2_ and NBT staining for $O_{2}^{-}$ under normal and chilling conditions. The result showed that OE plants accumulated a similar level of H_2_O_2_ and $O_{2}^{-}$ compared to WT plants before chilling treatment (Figure 8A). After chilling for 36 h, the leaves of OE plants accumulated more H_2_O_2_ and less $O_{2}^{-}$ compared to WT plants (Figure 8B). Consistently, more H_2_O_2_ and less $O_{2}^{-}$ were still detected in OE leaves compared to WT plants after chilling for 57 h (Figure 8C). These results indicate that OE lines accumulate more H_2_O_2_ than $O_{2}^{-}$ when compared with WT plants, which probably causes cell damage and impairs rice chilling tolerance.

### 2.7. The Delayed Induction of Antioxidant Enzymes Probably Leads to the Accumulation of H_2_O_2_ in OE Lines

**Figure 9:**
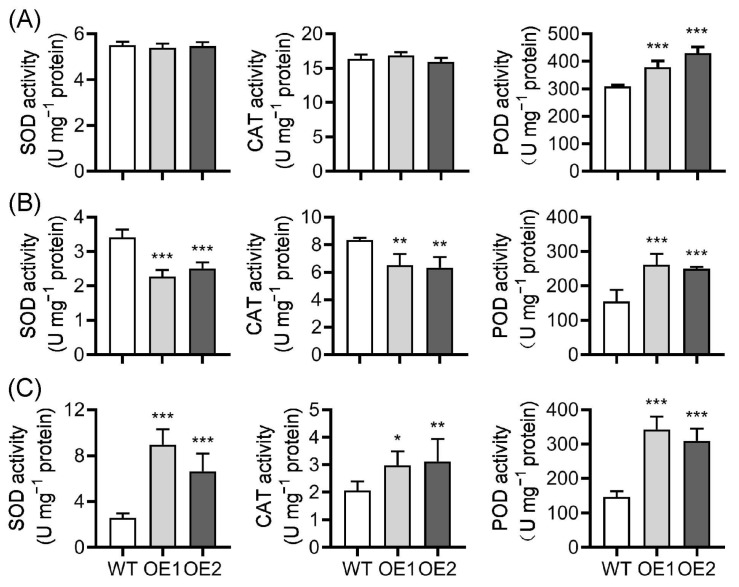
Analysis of antioxidant enzyme activities in wild-type (WT) and *OsPIN9*-overexpressing (OE) plants before and after chilling treatments. Enzyme activities were detected before chilling stress (**A**) and after chilling for 36 h (**B**) and 57 h (**C**) in WT and OE lines. Values are means ± standard deviation (SD) (*n* = 6). The data were analyzed by ANOVA and Tukey’s tests at a *p* < 0.05 significance level. *: *p* < 0.05; **: *p* < 0.01; ***: *p* < 0.001.

ROS are vital signaling molecules that play a crucial role in biotic and abiotic stress responses, while their content should be carefully controlled in plant cells via an array of enzymatic and non-enzymatic antioxidants [23]. Given that ROS homeostasis is disturbed in OE lines, we then analyzed the activities of three antioxidant enzymes, including superoxide dismutase (SOD), catalase (CAT), and peroxidase (POD), which play a key role in ROS scavenging [10,23], before chilling treatment and after chilling for 36 h and 72 h. There were no significant differences in SOD and CAT activities in WT and OE plants, while POD activity was significantly increased in OE lines compared to WT plants under normal conditions (Figure 9A). After chilling for 36 h, the activities of CAT and SOD decreased significantly in OE plants compared to those in WT plants. To our surprise, the POD activity was still strikingly higher in OE lines than in WT plants (Figure 9B). After chilling for 57 h, the activities of the three enzymes in OE lines all increased greatly compared to those in WT plants (Figure 9C). These results imply that it is likely that the decreased activities of SOD and CAT rather than increased POD activity led to the accumulation of H_2_O_2_ in OE lines at the early chilling stage, and the delayed induction of SOD and CAT is ineffective to quench excess ROS at the late chilling stage and causes cell damage in OE lines. However, considering that SOD usually functions in the conversion of $O_{2}^{-}$ to H_2_O_2_, the source of the accumulated H_2_O_2_ during chilling stress needs further investigation.

### 2.8. OsRobh Genes Show Differential Expression in OsPIN9-Overexpressing Lines

**Figure 10:**
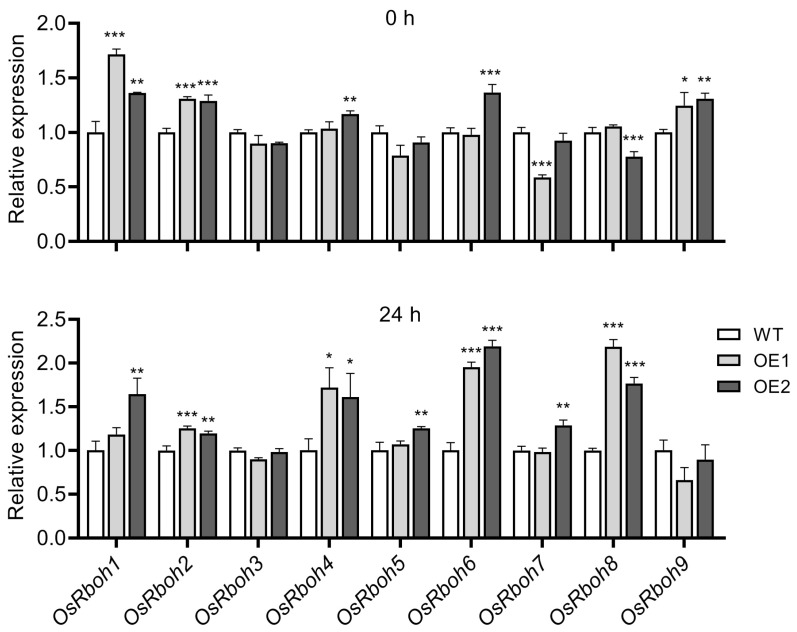
*OsRboh* expression analysis in wild-type (WT) and *OsPIN9*-overexpressing (OE) lines before and after chilling treatment. The expression level of *OsRboh* genes in WT was set to one. The data were analyzed by ANOVA and Tukey’s tests at a *p* < 0.05 significance level. *: *p* < 0.05; **: *p* < 0.01; ***: *p* < 0.001.

Previous reports showed that respiratory burst oxidase homologs (Rboh), also known as NADPH oxidase, play critical roles in various cellular activities and responses to abiotic stresses by regulating the production of ROS [70,71,72]. For the higher production of H_2_O_2_ detected in OE lines, we assayed the expression of *OsRboh* genes before and after chilling stress treatment to evaluate the source of H_2_O_2_ in OE lines. As shown in Figure 10, under normal conditions, most *OsRboh* genes in transgenic lines kept similar expression levels compared to those in WT plants, except for *OsRboh1*, *OsRboh2*, and *OsRboh9*, which showed a slight increase in OE lines. After chilling for 24 h, more *OsRboh* genes were induced, including *OsRboh2*, *OsRboh4*, *OsRboh6*, *and OsRboh8*, and showed a relative higher expression level in OE lines, which may contribute to the production of H_2_O_2_ in transgenic lines under chilling conditions.

## 3. Discussion

Although excellent progress has been made in the molecular mechanisms of plant cold adaptation [8,9,10], auxin, the first phytohormone discovered and one of the most important phytohormones in plant growth and developmental regulation, its role in modulating chilling adaptation is still elusive. In this present study, the role of a monocot-specific *OsPIN9* gene, which mainly functions in regulating auxin transport basipetally, in chilling stress was investigated by overexpression technology. 

Auxin plays a vital role in regulating almost all cellular processes, and in many cases, an appropriate auxin level and distribution within plant tissues are direct determinators affecting plant architecture [73]. *PIN* genes have been demonstrated to play a central role in auxin efflux and, consequently, influence auxin content and distribution in various tissues. Expression levels of *PIN* genes are usually closely associated with plant architecture [74,75]. The most representative is *AtPIN1*, and disruption of *AtPIN1* seriously influences inflorescence development and leads to the formation of needle-like inflorescence in *Arabidopsis* [76]. The rice genome possesses 12 *OsPIN* genes [45,46], and although the possibility of redundant functions of *OsPIN* genes exists [49], mutation of most *OsPIN* genes substantially influences rice architecture [47,49,53,54,55,77]. The previous report demonstrated that the OsPIN9 carrier functions in auxin transport basipetally, overexpression of *OsPIN9* substantially increases auxin content in rice roots [55], so we speculate that the auxin content in OE plant roots should be increased compared to that in WT plants. In line with this, the OE plant roots displayed a more resistant phenotype under NPA treatment (Figure 4), implying an increased auxin content in the OE roots. It was reported that overexpression of *OsPIN9* increased the adventitious root number [55]. Unexpectedly, we observed a lower number of adventitious roots in these two OE lines, which is different from the previous report [55]. Considering that adventitious root number is tightly associated with auxin content [55], and auxin regulates plant growth and development usually in a concentration-dependent manner, relative high auxin content accelerates plant growth, while excess auxin content strongly inhibits plant growth. Therefore, we speculate that the dramatic expression of *OsPIN9* probably leads to excess accumulation of auxin in OE roots, which suppresses adventitious root number (Figure 3). In agreement with this, the relative lower expression of *OsPIN9* (A2 line) significantly increased adventitious root number when compared to WT plants (Figure S1).

Rice is sensitive to chilling stress [7,78,79,80], and severe chilling disasters hamper rice growth and strikingly impair rice productivity [10]. Previous reports have suggested that chilling stress is implicated in auxin transport in plants [29,30,61,62]. However, there is no direct genetic evidence regarding the role of auxin transport in the regulation of chilling stress. In this study, we provided direct evidence that OsPIN9, which functions in auxin transport basipetally and could be quickly suppressed by chilling stress (Figure 1), is involved in rice chilling tolerance. The chilling treatment assay showed that OE lines are more sensitive to low temperatures than WT plants (Figure 5); further experiments confirmed that OE lines possess more dead cells (Figure 6A), higher membrane permeability (Figure 6B), and increased MDA content (Figure 6C) compared to WT plants after chilling treatment. Increased MDA content usually acts as a marker of lipid peroxidation [81], especially under various abiotic stresses [82,83], including chilling stress. Apart from rice, MDA levels were also employed to evaluate chilling tolerance in other plants, such as bermudagrass (*Cynodon dactylon*) [84] and *Pyrus betulaefolia* [85]. Collectively, these results strongly demonstrate that *OsPIN9* is involved in regulating rice chilling tolerance, which is probably mediated by influencing auxin transport. Over the last decade, significant progress has been made in the dissection of the underlying molecular mechanisms of plant chilling adaptation. At least the CBF/DREB-dependent cold-signaling pathway, the Ca^2+^ signaling pathway, the ROS-dominated adaptation mechanism, and the phytohormone regulation mechanism [1,9,86] have been demonstrated to regulate plant cold adaptation. Several lines of evidence have strongly suggested that overexpression of *OsPIN9* notably impairs rice chilling tolerance (Figure 5 and Figure 6), while how *OsPIN9* influences plant chilling tolerance is completely unknown. To this end, we first examined whether the impaired chilling tolerance of OE lines is associated with the CBF/DREB-dependent cold signaling pathway and the Ca^2+^ signaling pathway by employing qRT-PCR technology. The expression of *OsDREB1A*, *OsDREB1B*, and *OsDREB1C* was detected before and after chilling stress due to the sharp induction of these genes under chilling conditions [19,67]. Surprisingly, the *OsDREB1* genes, *OsTPP1*, *COLD1*, and *OsCNGC9* were all induced before treatment and kept at similar or even lower levels in OE lines compared to those in WT plants after chilling treatment (Figure 7). Considering that *OsDREB1* genes are mainly induced by low temperatures [19], and higher expression of *OsTPP1*, *COLD1*, and *OsCNGC9* also facilitates plant cold adaptation [7,14,68], we speculate that a stress response probably occurred in OE lines before chilling treatment. In contrast, all genes show a similar or lower expression level in OE lines compared to WT plants under chilling conditions, suggesting that the DREB regulon and Ca^2+^ signaling might be implicated in the impaired chilling tolerance of OE lines. Further investigations are needed to address this speculation. 

Next, we assayed ROS levels to clarify the role of ROS homeostasis in WT and OE lines under chilling stress. Chilling stress triggers ROS accumulation [21,87]. Low-level H_2_O_2_ can act as a signal to trigger the stress response at the early stress stage, whereas at the later stress stage, high-level H_2_O_2_ can damage plant cells [10,87]. Surprisingly, we only observed the accumulation of H_2_O_2_ in OE leaves at both early and late chilling stages, while $O_{2}^{-}$ was not accumulated during the chilling stages (Figure 8), indicating that ROS homeostasis is disturbed in OE plant cells and that H_2_O_2_ rather than $O_{2}^{-}$ causes cell damage in OE lines. It has been demonstrated that ROS homeostasis plays a vital role in plant abiotic stress adaptations [88,89], and ROS content must be carefully controlled by many ROS scavenging and detoxification systems during stress conditions [10]. Antioxidant enzyme activity measurement showed that SOD and CAT activities were significantly decreased and increased in OE lines compared to those in WT plants at the early and late chilling stages, respectively, while POD activity was always strikingly higher than that in WT plants (Figure 9B,C), even before chilling treatment (Figure 9A), indicating that the prompt trigger of antioxidant enzymes, mainly SOD and CAT in OE lines, plays a vital role in quenching accumulated ROS at the early chilling stage. Additionally, although antioxidant enzymes showed significantly higher activities in OE lines than in WT plants at the late chilling stage (Figure 9C), which could not detoxify excess H_2_O_2_ quickly (Figure 8C), indicating that the delayed induction of the antioxidant enzymes is not enough to scavenge ROS at the late chilling stage, and the constantly high levels of H_2_O_2_ then damage cells. 

To further evaluate the source of H_2_O_2_ in OE lines under chilling conditions, we assayed the expression of *OsRboh* genes before and after chilling stress. Since only a slight increase of *OsRboh1*, *OsRboh2*, and *OsRboh9* was detected in OE lines before chilling stress, it is reasonable that less ROS accumulation was observed under normal conditions. In contrast, more *OsRboh* genes were induced after chilling treatment, indicating that *OsRboh* is at least in part involved in ROS production after chilling stress. We also noticed that these induced *OsRboh* genes were still kept at low expression levels (up to about twofold) even after chilling treatment (Figure 10). Although evidence has shown that *Rboh* plays a vital role in ROS production under various abiotic stress conditions [70,71], we cannot rule out the possibility that other ROS-production sources probably exist that contribute to the ROS burst after chilling stress in OE lines.

## 4. Materials and Methods

### 4.1. Plant Materials, Growth Conditions, and Chilling Treatment

Rice *japonica* variety Nipponbare was employed for the physiological experiments and rice transformation. Hydroponic experiments were performed according to our previous report [90]. Briefly, rice seeds were sterilized first and then cultured in darkness for 3–4 days at 30 °C. The germinated seeds were transferred to Kimura B complete nutrient solution in plant growth chambers with a 12 h of light (30 °C)/12 h of darkness (25 °C) photoperiod and 60–70% relative humidity. 

To assay the responses of *OsPIN* genes to chilling treatment, 14 day old seedlings were transferred to low temperatures (4 °C), and root samples were collected at the indicated time points. The samples were then frozen quickly using liquid nitrogen and preserved at −80 °C for gene expression analysis.

To evaluate rice chilling tolerance, 14 day old WT and OE seedlings were transferred to 4 °C for 36 h and 57 h, followed by a 3 d recovery, and then the survival rate and physiological indicators were analyzed.

### 4.2. Vector Construction and Generation of the Transgenic Plants

The full-length *OsPIN9* gene was amplified by PrimeSTAR HS DNA Polymerase (TaKaRa Biotechnology Co., Ltd., Dalian, China) from rice cDNA using the specific primers listed in Table S1. A plant expression vector, pCAMBIA1301-pUbi, which was kindly provided by Dr. Yao-Guang Liu (College of Life Sciences, South China Agricultural University, Guangzhou, China), was used for overexpressing *OsPIN9*. To insert into the expression vector pCAMBIA1301-pUbi, the restriction sites *Bam*H I and *Kpn* I were added at the 5′ end of forward and reverse primers, respectively. The recombinant construct was named *pUbi*:*OsPIN9* and confirmed by restriction enzyme digestion and sequencing. The constructed plasmid was then transformed into *Agrobacterium tumefaciens* (EHA105) and employed for rice transformation according to the previous report [91]. Homozygous T_3_-generation transgenic plants were used for further studies. 

### 4.3. Quantitative RT-PCR

qRT-PCR was performed according to a previous report [39]. In short, total RNA was extracted from the collected samples using RNAiso Plus (TAKARA Bio Inc., Dalian, China), and reverse transcription was conducted using HiScript III RT SuperMix for qPCR (Nanjing Vazyme Biotech Company, Ltd., Nanjing, China). The online website INTEGRATED DNA TECHNOLOGIES (https://sg.idtdna.com, accessed on 1 May 2019) was used to design the gene-specific primers. qRT-PCR was conducted using AceQ Universal SYBR qPCR Master Mix (Nanjing Vazyme Biotech Company, Ltd.) and the Lightcycle^®^ 96 system. At least three biological replicates and three technical repetitions were performed to assay the gene expression. The *OsACTIN1* gene (Os03g0718100) was employed as an internal control. All primers used in this study are listed in Table S1, and gene names and ID numbers used for qRT-PCR in this study are listed in Table S2. 

### 4.4. Exogenous NPA Treatment

NPA is usually employed as a classical inhibitor of polar auxin transport to elucidate the underlying mechanisms of plant growth and development associated with polar auxin transport [65]. For analyzing the responses of WT and OE lines to NPA treatment, germinated seeds were transferred into Kimura B complete nutrient solution containing 0.5 μM NPA and cultured for 7 d, and then the shoot height, root length, and adventitious root number were assessed. WT and OE lines cultured under normal conditions were used as controls in this experiment.

### 4.5. Physiological Analysis

Trypan blue staining was conducted as described previously [92] with minor modifications. Rice leaves were sampled before and after chilling treatment and stained with a lactophenol-trypan blue solution (10 mL lactic acid, 10 mL glycerol, 10 g phenol, and 10 mg trypan blue, mixed in 10 mL distilled water). The leaves were boiled in a water bath for 10 min in trypan blue solution and then kept at room temperature for 1 h. Decolorization was performed using chloral hydrate solution (25 g chloral hydrate dissolved in 10 mL distilled water). Electrolyte conductivity and MDA content were detected according to the previous report [93]. 3′3-diaminobenzidine (DAB) and nitro blue tetrazolium (NBT) staining were performed as previously reported [21]. Briefly, rice leaves were first cut into sections (about 2 cm in length) and then soaked in DAB and NBT solutions, respectively, overnight at 37 °C. Alcohol (95%) was used for decolorization. CAT, POD, and SOD activities were assayed according to the previous methods [94] with minor modifications. In short, 0.1 g of rice leaves were broken with liquid nitrogen and then homogenized in 1 mL of extraction buffer (50 mM phosphate, pH 7.8). After centrifugation, the supernatant was used for enzyme activity assays. CAT activity was measured in a reaction solution consisting of 50 mM phosphate buffer (pH 7.0), 0.2% H_2_O_2_, and enzyme extract. POD activity was measured in a reaction mixture (0.2% H_2_O_2_, 0.2% guaiacol, and enzyme extract) at 470 nm. SOD activity was assayed in a reaction mixture containing 50 mM phosphate, pH 7.8, 130 mM L-methionine, 750 μM NBT, 100 μM EDTA, 20 μM riboflavin, and enzyme extract at 560 nm. The Coomassie Brilliant Blue G-250 staining method was employed to measure protein content [95].

### 4.6. Data Analysis

Each experiment was biologically replicated at least three times. The one-way analysis of variance (ANOVA) method was used to statistically analyze experimental data by GraphPad PRISM 8 version 8.0.2 (GraphPad Software Inc., San Diego, CA, USA) at the significance levels of *p* < 0.05 (*), *p* < 0.01 (**), and *p* < 0.001 (***), and all data are given as means ± SD.

## 5. Conclusions

In conclusion, in this report, we systematically monitored the responses of *OsPIN* genes to chilling treatment and generated transgenic rice plants with high expression levels of *OsPIN9*. The transgenic plants showed impaired chilling tolerance compared to WT plants, which is probably mainly caused by the sharp accumulation of H_2_O_2_ and delayed induction of SOD and CAT. *OsRboh* genes, at least in part, contribute to the production of ROS in OE lines under chilling stress conditions. The DREB regulon and Ca^2+^ signaling pathway are implicated in the chilling tolerance of OE lines, while the detailed functional mechanisms need further investigation. Several issues need further discussion, for example, how the relationship between auxin homeostasis and ROS homeostasis is derived and where the accumulated H_2_O_2_ is derived from? What is the role of $O_{2}^{-}$ and POD in OE lines under chilling stress? Further investigation focused on these detailed issues may lead to insight into the underlying molecular mechanisms of rice chilling tolerance and may provide potential targets for breeding chilling-resistant crops.

## Data Availability

Not applicable.

## Supplementary Materials

The following supporting information can be downloaded at: https://www.mdpi.com/article/10.3390/plants12152809/s1, Figure S1: Adventitious root number analysis in the transgenic A2 line; Table S1: Primers used in this study; Table S2: Gene names and ID numbers used for qRT-PCR in this study.
