# Supplementary material for: Overexpression of OsPIN9 Impairs Chilling Tolerance via Disturbing ROS Homeostasis in Rice
Source: Plants (Basel). 2023 Jul 28;12(15):2809. doi: 10.3390/plants12152809 (PMC10421329; doi:10.3390/plants12152809)

Figure S1. Adventitious root number analysis in transgenic A2 line. Values are means ± standard deviation (SD) (n = 20). The data were analyzed by ANOVA and Tukey's tests at a $p < 0.05$ significance level. *: $p < 0.05$; **: $p < 0.01$.

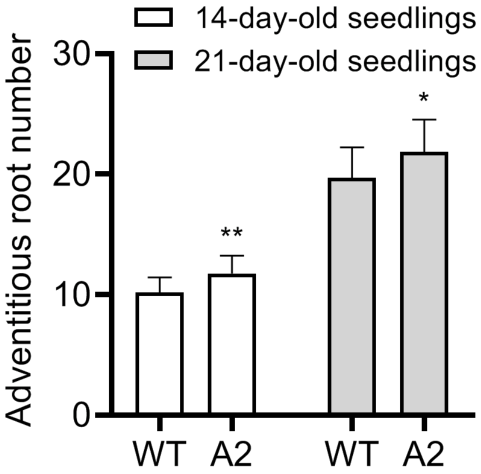

Supplement: Supplementary file 1 [file plants-12-02809-s001.zip › Supplementary files-Figure S1.pdf]
