# Supplementary material for: Overexpression of OsPIN9 Impairs Chilling Tolerance via Disturbing ROS Homeostasis in Rice
Source: Plants (Basel). 2023 Jul 28;12(15):2809. doi: 10.3390/plants12152809 (PMC10421329; doi:10.3390/plants12152809)
Supplement: Supplementary file 1 [file plants-12-02809-s001.zip › Supplementary files-Table S1.pdf]

Table S1. Primers used in this study.

**Primers for vector construction**	Primer sequences (5′-3′)
OsPIN9-BamH1-F	TATAGGATCCATGATTACGGGTTCGGAGGT
OsPIN9-Kpn1-R	GTCAGGTACCCACAGCCCCAACAGAATATA
**Primers for screening of the transgenic plants**	
HPT-F	CTGAACTCACCGCGACGTCTGTC
HPF-R	TAGCGCGTCTGCTGCTCCATACA
**Primers for qRT-PCR**	
OsPIN1b-qF	GAATCGTGCCCTTTGTGTTTG
OsPIN1b-qR	TGTAGTAGACGAGGGTGATAGG
OsPIN1c-qF	GAGCAATCAGCATCCCGAATA
OsPIN1c-qR	GAGCAATCAGCATCCCGAATA
OsPIN1d-qF	TCAGGAACCCAAACACTTACTC
OsPIN1d-qR	TGATAGCCGGCATCTCAATTC
OsPIN2-qF	CGTCTCCTTCAGGTGGAATATC
OsPIN2-qR	AGAGCCATGAACAAGCCTAAG
OsPIN5a-qF	CCCTACCTCAATCCATCACATC
OsPIN5a-qR	GTAGGGAGACAAGCATTCCAA
OsPIN5c-qF	GACACAAGTCCTCACGATGAA
OsPIN5c-qR	TAACCGCTGTGCTGAGTATTT
OsPIN9-qF	GAGGACTCTCTGTTCACCATTC
OsPIN9-qR	GAGAACGACGCTATCTTGTATCC
OsPIN10a-qF	GTCGAGAAGTCCATCTCCATTC
OsPIN10a-qR	TTGCCACACGCGATGAT
OsPIN10b-qF	CTTGCGTATCGCCATTGTTC
OsPIN10b-qR	GGTAGCGTGGAGGTTGTATT
OsDREB1A-qF	AGCGACCTGGCGTTCG
OsDREB1A-qR	TCGCGTAGTACAGGTCCCA
OsDREB1B-qF	GAGACCTTCGCCAACGATG
OsDREB1B-qR	CACCGGCAACACGTCCTT
OsDREB1C-qF	TACGGCAACATGGACTTCGA
OsDREB1C-qR	GCCCATCCCGTCGTAGTAGTAG
OsTPP1-qF	TGTCTCCCGTGATGAGAGCTG
OsTPP1-qR	AAACACCTTATTGCGGGACCTT
OsCNGC9-qF	GTGCTGTTTCTGCTCCATTTC
OsCNGC9-qR	TGCACTTGTCTGAAGAGGATTT
COLD1-qF	CAGGATATCAAAAGCTTGGATG
COLD1-qR	GCAGCTATCTTTGCTTGACG
OsRboh1-qF	AGAACTGTTTTCTCTGAGGC
OsRboh1-qR	AAGTTTTGGGAATCTTGCTT
OsRboh2-qF	GAAATGCACAACGAAGTTCGAA

OsRboh2-qR	TAACTATATGCTTGCTTCCTCA
OsRboh3-qF	TACACTCACGAAACAACTCAAGG
OsRboh3-qR	TATTTGCTATCCCAATGCTATCGT
OsRboh4-qF	GCAGCAAGATCCGGACACAT
OsRboh4-qR	GAACCGAGTCGTTGTCGTCT
OsRboh5-qF	CAGATGCACGTCAAAATTTGAG
OsRboh5-qR	CTGAGTATCTCAAGCAAGAAGT
OsRboh6-qF	AAAGCCCACACTAGCCAAAGAAT
OsRboh6-qR	AGAGGGTCAGTTTGCTTCTATGC
OsRboh7-qF	AACAGTTGGAGTTTTCTACTGC
OsRboh7-qR	TGGAGATCAGGAGAAGCTATATC
OsRboh8-qF	AGCTCGCTCAAGATTTCTCGAG
OsRboh8-qR	TTGATCTAATCCCTCTACATTTCCA
OsRboh9-qF	TCATCGGATTCATCGTCGTC
OsRboh9-qR	CGAACAACCAATCACTCACT
OsACTIN1-qF	CTTCATAGGAATGGAAGCTGCG
OsACTIN1-qR	CACCTTGATCTTCATGCTGCTA
