# Supplementary material for: Overexpression of OsPIN9 Impairs Chilling Tolerance via Disturbing ROS Homeostasis in Rice
Source: Plants (Basel). 2023 Jul 28;12(15):2809. doi: 10.3390/plants12152809 (PMC10421329; doi:10.3390/plants12152809)
Supplement: Supplementary file 1 [file plants-12-02809-s001.zip › Supplementary files-Table S2.pdf]

Table S2. Gene names and ID numbers used for qRT-PCR in this study.

Gene name	Gene ID
OsPIN1b	Os02g0743400
OsPIN1c	Os11g0137000
OsPIN1d	Os12g0133800
OsPIN2	Os06g0660200
OsPIN5a	Os01g0919800
OsPIN5c	Os09g0505400
OsPIN9	Os01g0802700
OsPIN10a	Os01g0643300
OsPIN10b	Os05g0576900
OsDREB1A	Os09g0522200
OsDREB1B	Os09g0522000
OsDREB1C	Os06g0127100
OsTPP1	Os02g0661100
OsCNGC9	Os09g0558300
COLD1	Os04g0600800
OsRboh1	Os01g0360200
OsRboh2	Os01g0734200
OsRboh3	Os01g0835500
OsRboh4	Os05g0465800
OsRboh5	Os05g0528000
OsRboh6	Os08g0453700
OsRboh7	Os09g0438000
OsRboh8	Os11g0537400
OsRboh9	Os12g0541300
OsACTIN1	Os03g0718100
